# The Diagnostic Value of Artificial Intelligence Ultrasound S-Detect Technology for Thyroid Nodules

**DOI:** 10.1155/2022/3656572

**Published:** 2022-11-26

**Authors:** Peizhen Huang, Bin Zheng, Mengyi Li, Lin Xu, Sajjad Rabbani, Abdulilah Mohammad Mayet, Chengchun Chen, Beishu Zhan, He Jun

**Affiliations:** ^1^Department of Ultrasound and Imaging, Wenzhou Central Hospital, Wenzhou 325000, China; ^2^Wenzhou Medical University, Wenzhou 325000, China; ^3^Department of Electrical Engineering, Lahore College for Women University, LCWU, Lahore, Pakistan; ^4^Electrical Engineering Dept, King Khalid University, Abha 61411, Saudi Arabia

## Abstract

This study aimed to evaluate the consistency of ultrasound TI-RADS classification used by sonographers with different ultrasound diagnosis experience in the diagnosis of thyroid nodules and the diagnostic value of using artificial intelligence ultrasound S-Detect technology in the differentiation of benign and malignant thyroid lesions. 100 patients who underwent ultrasound examination of thyroid masses in our hospital from June 2019 to June 2021 and were further punctured or operated on were included in the study. Pathological results were used as the gold standard to evaluate ultrasound S-Detect technology and the value of TI-RADS classification and the combined application of the two in diagnosing benign and malignant thyroid TI-RADS 4 types of nodules, and the consistency of judgments of doctors of different ages is assessed by a Kappa value. There were 128 nodules in 100 patients, 51 benign nodules, and 77 malignant nodules. For senior physicians, the sensitivity of diagnosis using TI-RADS classification combined with ultrasound S-Detect technology is 93.5%, specificity is 94.1%, and accuracy is 93.8%; for middle-aged physicians using TI-RADS classification combined with ultrasound S-Detect technology for diagnosis, the sensitivity is 89.6%, specificity is 92.2%, and accuracy is 90.6%; for junior doctors, the sensitivity of diagnosis using TI-RADS classification combined with ultrasound S-Detect technology is 83.1%, specificity is 88.2%, and accuracy is 85.1%. Regardless of seniority, the combined application of artificial intelligence ultrasound S-Detect technology and TI-RADS classification can improve the diagnostic ability of sonographers for thyroid nodules and at the same time improve the consistency of judgment among physicians, and this is especially important for radiologists.

## 1. Introduction

Athyroid nodule is one of the most common nodular lesions in adults, and its incidence is getting higher and higher. The prevalence rate of Chinese residents is as high as 18.6%, and it is more common in women, and most of them are benign nodules, only 7% of thyroid nodules tend to be malignant, but 5% of patients will change from benign to malignant without timely diagnosis and treatment [1]. The incidence of thyroid cancer increases with age, and it is the malignant tumor with the largest number of new patients among all cancer types in recent years [2]. According to statistics from the National Cancer Research Center of the United States, there were about 64,300 new cases of thyroid cancer worldwide in 2016, and about 1,980 people died of thyroid cancer [3]. Timely and accurate detection of thyroid nodules and identification of benign and malignant thyroid nodules are of great significance to improve clinical treatment effects and prognosis of patients [4].

Fine-needle aspiration biopsy (FNAB) or surgical biopsy is the gold standard for diagnosing thyroid cancer, while for incidentally discovered thyroid nodules, ultrasound is the most commonly used noninvasive method to differentiate benign and malignant thyroid nodules [5]. Ultrasound is a real-time imaging method, which has the advantages of no radiation and no damage, high accuracy, simple and fast operation, and low cost, but it is highly dependent on the operator and has a low repetition rate of diagnostic results [6]. With the development and application of artificial intelligence (AI) technology in the medical field, the AI ultrasound intelligent auxiliary diagnosis system based on static ultrasound images has emerged, which can greatly reduce the labor of medical workers while ensuring accuracy. However, the collection of static ultrasound images is still affected by multiple factors, while dynamic AI can perform real-time synchronous dynamic analysis of nodules from multiple levels and angles, determine the nature of nodules, and further improve diagnostic efficiency. There is no domestic report on the dynamic AI ultrasound intelligently assisted real-time diagnosis system [7–11]. The application of computer-aided diagnosis (CAD) technology “S-Detect^TM^” can realize the qualitative and quantitative automatic analysis of ultrasound images and obtain objective, repeatable, and more accurate diagnostic results. In the latest generation of ultrasonic diagnostic equipment, S-Detect^TM^ technology adopts the method of deep learning, which can improve the accuracy of diagnosis. At present, only a few scholars have reported the preliminary application of S-Detect^TM^ technology in the differential diagnosis of thyroid masses [9–14]. The purpose of this study was to investigate the consistency of ultrasound TI-RADS classification in the diagnosis of thyroid nodules by sonographers with different ultrasound diagnostic experiences, to explore the diagnostic value of using artificial intelligence ultrasound S-Detect technology in the differentiation of benign and malignant thyroid lesions, as well as the clinical value of the combined diagnosis of S-Detect technology and routine thyroid ultrasonography, so as to provide sonographers with a more objective and accurate assessment tests for thyroid tumors.

## 2. Research Proposal and Object

### 2.1. Research Objects

A total of 100 patients who underwent ultrasound examination of thyroid masses in our hospital from June 2019 to June 2021 and underwent further puncture or surgery were included in the study.

#### 2.1.1. Inclusion Criteria

  The patient had one or more thyroid nodules
  The patient was about to undergo FNAB or surgery
  Aged ≥ 18 years old
  The patient signed informed consent

#### 2.1.2. Exclusion Criteria

Exclusion criteria were as follows: patients with diseases that are unfavorable to the trial or pose a threat to other participants, such as mental illness; unable to cooperate with the trial operation; pregnant or breastfeeding women; poor quality of ultrasound images that cannot meet parameter measurement and analysis; history of thyroid surgery or history of thyroid biopsy; received radiotherapy and chemotherapy; no biopsy or surgical pathology results after examination; no clear diagnosis of benign and malignant after biopsy or surgery; simple cystic nodule, calcification; the tumor boundary that could not be identified; the underlying data were incomplete. This study was approved by the hospital's ethics committee (K20180216), and patients signed informed consent.

### 2.2. Instruments and Methods

We used SamsungRS80A (South Korea, Seoul, Samsung Madison Co., Ltd., L3-12A linear array high-frequency probe, frequency 5–13 MHz, equipped with S-Detect intelligent detection system) high-end color Doppler ultrasound diagnostic apparatus to scan the thyroid, to understand the overall situation of the thyroid, longitudinal and transverse scanning of nodules (select 2-3 nodules with pathological results and the most suspicious of malignancy) and then analyzed the color Doppler signal of the nodule, longitudinal section and transverse section of the nodule were saved. We entered the S-Detect mode, started the S-Detect automatic analysis program to automatically draw the region of interest (ROI) on the largest longitudinal section and the largest transverse section of the thyroid nodule, and output the S-Detect diagnostic report, and the ultrasound images and evaluation report records automatically analyzed by S-Detect were stored on the hard disk for later data sorting and data analysis.

### 2.3. Analytical Method

#### 2.3.1. Thyroid Nodule Ultrasound TI-RADS Classification Evaluation

All cases were routinely examined by two senior doctors with more than 10 years of experience in thyroid ultrasound diagnosis. Thyroid ultrasound TI-RADS classification assessment was performed by 6 sonographers with different experience in low, medium, and high grades and divided into three groups (there are 2 people in the low-level group, divided into low-level A and low-level B, physicians with 2 years of experience in thyroid disease ultrasound diagnosis, 2 people in the middle-level group, divided into middle-level A and middle-level senior B, physicians with 5 years of experience in thyroid disease ultrasound diagnosis, and there are 2 seniors in the senior group, divided into senior A and senior B, physicians with 10 years of experience in thyroid disease ultrasound diagnosis). According to the 2017 version of the ultrasound TI-RADS classification criteria [13], a double-blind method was used to evaluate the TI-RADS classification of thyroid nodules. The diagnostic cutoff between TI-RADS4 and TI-RADS5 was used as the diagnostic cut-off point for benign and malignant tumors, and TI-RADS4 was assessed as a possible benign tumor and TI-RADS5 was assessed as a possible malignant tumor. All sonographers were unaware of the clinical information of the cases, the number of benign and malignant cases, and the final diagnosis of the cases.

#### 2.3.2. Evaluation of Artificial Intelligence Ultrasound S-Detect Technology

We activate the S-Detect automatic analysis program, make precise identification of lesions based on various characteristics of lesions, and use the ultrasonic TI-RADS classification report to issue recommended diagnostic results for thyroid nodules, which are automatically generated S-Detect diagnostic reports, judge the lesion as “probably benign” or “probably malignant” and prompt the result of “binary classification,” and finally keep a record of images and evaluation results. If the prompting results of the two sections are different, it is regarded as “probably malignant.”

#### 2.3.3. Combined Diagnostic Method

If the classification results of artificial intelligence ultrasound S-Detect technology and the sonographer's BI-RADS classification assessment are inconsistent, when artificial intelligence ultrasound S-Detect technology diagnoses lesions as “possibly benign,” the original sonographer's TI-RADS classification diagnostic results drops down one level; when artificial intelligence ultrasound S-Detect technology diagnoses lesions as “possibly malignant,” the original sonographer's conventional two-dimensional ultrasound TI-RADS classification diagnostic result is upgraded by one level. When the original sonographer's conventional two-dimensional ultrasound TI-RADS classification diagnostic result is category 3, the combined diagnostic result does not decrease. All judgment results are compared with the results of puncture or surgical pathology. With the results of puncture biopsy or surgical pathology as the gold standard, the ultrasound TI-RADS 3–5 results of physicians with different seniority levels are transformed into a benign and malignant dichotomous model, and with BI-RADS category 4 being the cutoff point for benign and malignant, possibly benign includes categories 1, 2, 3, and 4 and possibly malignant includes category 5.

### 2.4. Statistical Analysis

After sorting out all the statistical results, a database was established, and SPSS (SPSS19.0, IBM) and MedCalc19.0 statistical analysis software was used for data analysis. Linear weighted kappa coefficient analysis was used to calculate the Kappa value to evaluate the consistency of ultrasound TI-RADS classification in evaluating thyroid nodules among physicians of different seniority levels. The 2 ∗ 2 contingency table and the chi-square test were used to calculate the sensitivity, specificity, and accuracy of different senior doctors applying TI-RADS classification, artificial intelligence ultrasound S-Detect technology, and the combination of different senior doctors and S-Detect technology in differential diagnosis of benign and malignant thyroid lesions. We drew the ROC characteristic curve of different senior physicians using ultrasonic TI-RADS classification, artificial intelligence ultrasound S-Detect technology, and the combined diagnosis of thyroid lesions by different senior physicians and S-Detect technology and calculated the area under the ROC characteristic curve (AUC). The *Z* test was used to compare the differences in AUC of different inspection methods. The enumeration data were expressed as a percentage, and the chi-square test of paired data was used to compare the between-group comparison between the ultrasonic features of thyroid tumors judged by artificial intelligence ultrasound S-Detect technology and the results judged by sonographers. When *P* < 0.05, the difference was statistically significant. Consistency assessment: 0.6–0.8 is fair, 0.8–0.9 is good, and 0.9–1.0 is excellent.

## 3. Results

### 3.1. Pathological Result

There were 128 nodules in 100 patients (among patients, 37 were female and 63 were male), of which 79 were pathologically confirmed by surgical resection, 56 were pathologically confirmed by cytology or histological biopsy, 51 were benign (19 nodular goiters, 16 thyroid adenomas, 11 Hashimoto's thyroiditis, 5 nodular goiters with cystic degeneration), and 77 malignant nodules (43 papillary thyroid carcinoma, 22 follicular thyroid carcinoma, 10 Hashimoto's thyroiditis complicated with papillary thyroid carcinoma, and 2 medullary thyroid carcinoma).

### 3.2. Diagnostic Results of Senior Physicians and Different Diagnostic Methods

**Figure 1:**
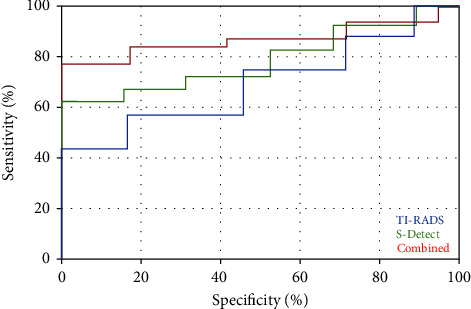
ROC curves of senior physicians using different diagnostic methods.

For senior physicians, 63 benign nodules and 65 malignant nodules were diagnosed by TI-RADS classification, 57 benign nodules and 71 malignant nodules were diagnosed by ultrasound S-Detect technology, and when the two were used together, 55 benign nodules and 73 malignant nodules were diagnosed, and the kappa value was between 0.6 and 1; the sensitivity, specificity, and accuracy of TI-RADS for diagnosing thyroid nodules were 83.1%, 88.2%, and 85.1%, respectively, and AUC = 0.796, and the diagnostic sensitivity of ultrasonic S-Detect technology was 89.6%, specificity was 88.2%, accuracy was 89.1%, and AUC = 0.869. When the two were combined, diagnostic sensitivity was 93.5%, specificity was 94.1%, accuracy was 93.8%, and AUC = 0.923 (Table 1, Figure 1).

### 3.3. Diagnostic Results of Middle-Aged Physicians and Different Diagnostic Methods

**Figure 2:**
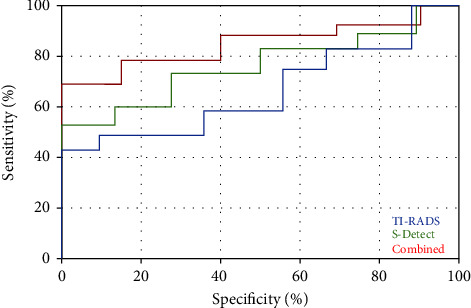
ROC curves of middle-aged physicians using different diagnostic methods.

For middle-aged physicians, 62 benign nodules and 66 malignant nodules were diagnosed by TI-RADS classification and 54 benign nodules and 74 malignant nodules were diagnosed by ultrasound S-Detect technology. When the two were used together, 53 benign nodules and 75 malignant nodules were diagnosed, and the kappa value was between 0.6 and 1; the sensitivity, specificity, and accuracy of TI-RADS for diagnosing thyroid nodules were 80.5%, 78.4%, and 80.4%, respectively, and AUC = 0.713, and the diagnostic sensitivity of ultrasonic S-Detect technology was 83.1%, specificity was 84.3%, accuracy was 83.6%, and AUC = 0.826. When the two were combined, diagnostic sensitivity was 89.6%, specificity was 92.2%, accuracy was 90.6%, and AUC = 0.897 (Table 2, Figure 2).

### 3.4. Diagnostic Results of Junior Physicians and Different Diagnostic Methods

**Figure 3:**
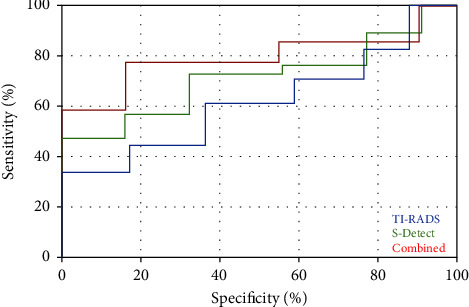
ROC curves of junior physicians using different diagnostic methods.

For junior physicians, 63 benign nodules and 65 malignant nodules were diagnosed by TI-RADS classification, 61 benign nodules and 67 malignant nodules were diagnosed by ultrasound S-Detect technology, and when the two were used together, 53 benign nodules and 75 malignant nodules were diagnosed, and the kappa value was between 0.4 and 0.8; the sensitivity, specificity, and accuracy of TI-RADS for diagnosing thyroid nodules were 72.7%, 76.5%, and 74.2%, respectively, and AUC = 0.685, the diagnostic sensitivity of ultrasonic S-Detect technology was 76.6%, specificity was 78.4%, accuracy was 77.3%, and AUC = 0.798. When the two were combined, diagnostic sensitivity was 83.1%, specificity was 88.2%, the accuracy was 85.1%, and AUC = 0.856 (Table 3, Figure 3).

## 4. Discussion

The combined application of medical image data and AI based on deep learning has revolutionized the expression of medical images. Many centers have carried out static AI diagnosis of thyroid nodules in clinical practice. A large number of research data at home and abroad show that AI is effective in judging benign and malignant thyroid nodules. It has high diagnostic value, is convenient and quick, and improves clinical examination and diagnostic efficiency [8–13]. On this basis, dynamic AI uses an ultra-large-scale convolutional neural network and deep learning technology to realize real-time localization, the real-time outline of thyroid nodules, and real-time auxiliary diagnosis of benign and malignant nodules during the inspection process, making ultrasonography more efficient and precise.

S-Detect^TM^ technology is an emerging computer-aided diagnostic method, based on Korea and Russ TI-RADS (Thyroid Image Reporting and Data System) classification and ATA (American Thyroid Association) guideline classification, and using the deep learning model CNN (convolutional neural network), it automatically detects and analyzes the internal structure, echo level, boundary, direction, shape, and other information of thyroid tumors to realize the diagnosis of benign and malignant lesions [15, 16]. The application of S-Detect^TM^ can not only improve diagnostic accuracy and repeatability but also greatly reduce the work pressure of ultrasound doctors, which has important reference value for beginners and clinicians.

Among the 128 nodules in the 100 patients in this study, 51 were benign nodules and 77 were malignant nodules confirmed by pathology. For senior physicians, the sensitivity, specificity, and accuracy of TI-RADS classification for diagnosing thyroid nodules were 89.6%, 89.6%, and 89.1%, respectively (kappa value 0.701); for middle-aged physicians, the sensitivity, specificity, and accuracy of TI-RADS classification for diagnosing thyroid nodules were 80.5%, 78.4%, and 80.4%, respectively (kappa value 0.604); for junior physicians, the sensitivity of TI-RADS classification for thyroid nodules was 72.7%, specificity was 76.5%, and accuracy was 74.2% (kappa value 0.440). With the increase in the physician seniority level, their diagnostic ability for thyroid nodules was significantly better, and they showed better evaluation consistency, confirming the influence of physician experience on the diagnosis of thyroid nodules.

Artificial intelligence ultrasound S-Detect technology is the first commercial image analysis program based on deep learning algorithms newly developed by Samsung Medical in recent years. It is installed on the high-end ultrasound diagnostic equipment of Samsung Madison in South Korea. The clinical application is mostly concentrated in the diagnosis of breast tumors [16–19], while the application in the diagnosis of thyroid nodules is less. In this study, the application of ultrasound S-Detect technology improved the specificity, sensitivity, and accuracy of thyroid nodule diagnosis among sonographers of different seniority, especially for middle and low-level doctors, and when ultrasound S-Detect technology is combined with the TI-RADS classification and the diagnostic method, it can significantly improve the diagnostic ability of middle and low-level physicians for thyroid nodules. This is especially important for junior physicians, because their diagnostic sensitivity for benign and malignant thyroid nodules using TI-RADS classification is only 72.7%, and the specificity (76.5%) and accuracy (74.2%) also need to be improved urgently. When the ultrasound S-Detect technique was used in combination with the TI-RADS classification, the diagnostic sensitivity of junior physicians for benign and malignant thyroid nodules increased to 83.1%, which was the same as that of senior physicians using the TI-RADS classification (88.2% specificity and 85.1% accuracy), at the same time, due to the application of artificial intelligence ultrasound S-Detect technology, the judgment of physicians tends to be correct, resulting in a significant improvement in the consistency of assessment among junior physicians, from general diagnostic consistency (kappa = 0.44) to stronger diagnostic consistency (kappa = 0.696), and this improvement in diagnostic consistency also occurred among middle-aged and senior physicians.

In conclusion, the results of this study show that regardless of their seniority, the combined application of artificial intelligence ultrasound S-Detect technology and TI-RADS classification can improve the diagnostic ability and diagnostic consistency of sonographers for thyroid nodules. It is especially obvious for junior doctors, which helps improve the diagnostic confidence of junior sonographers and avoid unnecessary needle biopsy or surgery, which is worthy of clinical promotion. However, this study also has certain limitations; for example, it is only a single-center retrospective study, and the number of patients is small.

## Figures and Tables

**Table 1:** Differences in diagnosis among senior physicians.

	TI-RADS classification		Total	Kappa	Ultrasound S-Detect		Total	Combined		Total	Kappa
	Benign	Malignant			Benign	Malignant		Benign	Malignant		
Benign	45	1	46	0.701	45	2	47	48	1	49	0.871
Malignant	18	64	82		12	69	81	7	72	79	
Total	63	65	128		57	71	128	55	73	128	
Specificity (%)	83.1				89.6			93.5			
Sensitivity (%)	88.2				88.2			94.1			
Accuracy (%)	85.1				89.1			93.8			

**Table 2:** Differences in diagnostic results among middle-aged physicians.

	TI-RADS classification		Total	Kappa	Ultrasound S-Detect		Total	Combined		Total	Kappa
	Benign	Malignant			Benign	Malignant		Benign	Malignant		
Benign	41	4	45	0.604	43	10	53	47	6	53	0.817
Malignant	21	62	83		11	64	75	8	69	75	
Total	62	66	128		54	74	128	53	75	128	
Specificity (%)	80.5				83.1			89.6			
Sensitivity (%)	78.4				84.3			92.2			
Accuracy (%)	80.4				83.6			90.6			

**Table 3:** Differences in diagnostic results among junior physicians.

	TI-RADS classification		Total	Kappa	Ultrasound S-Detect		Total	Combined		Total	Kappa
	Benign	Malignant			Benign	Malignant		Benign	Malignant		
Benign	39	9	68	0.440	40	8	48	45	11	56	0.696
Malignant	14	56	70		21	59	80	8	64	72	
Total	63	65	128		61	67	128	53	75	128	
Specificity (%)	72.7				76.6			83.1			
Sensitivity (%)	76.5				78.4			88.2			
Accuracy (%)	74.2				77.3			85.1			

## Data Availability

The data used to support the findings of this study are included within the article.
